# Knowledge, Attitude, and Practices Toward SARS-COV-2 Infection in the United Arab Emirates Population: An Online Community-Based Cross-Sectional Survey

**DOI:** 10.3389/fpubh.2021.687628

**Published:** 2021-07-19

**Authors:** Hamda Musabbah Alremeithi, Aljazia Khalfan Alghefli, Rouqyah Almadhani, Latifa Mohammad Baynouna AlKetbi

**Affiliations:** Al Ain-AHS Family Medicine Program-Academic Affairs, Abu Dhabi Healthcare Services, Abu Dhabi, United Arab Emirates

**Keywords:** SARS-CoV-2 infection, knowledge, attitude, practices, preventive measures

## Abstract

Population's preventive practices and self-isolation is determinantal in the prevention and mitigation. This study explored the adult population's knowledge, attitude, and practice toward COVID-19 in UAE between the 4th and 14th of April 2020. The study was a community-based, cross-sectional study using a self-administered electronic questionnaire covering five different aspects: demographics, knowledge, practice, attitude, source, and trust of information, and a patient health questionnaire (PHQ-2) for depression screening. Results were analyzed using frequencies, cross-tabulation, and regression analysis. A total of 1,867 people responded to the survey. The mean age of participants was 36.0 years S.D. 10.8. Males were 19.3% and female (80.7%). Knowledge was significantly better in people with higher educational levels (B 0.17, *P-value* < 0.001), good preventive practice (B 0.12, *P-value* < 0.001), and higher perceived risk scores (B 0.053, *P-value* = 0.025). The best practice scores were shown by participants with older age (B 0.097, *P-value* < 0.05), with good knowledge (B 0.086, *P-value* < 0.05), were of non-UAE nationalities (B −0.08, *P-value* < 0.05), with jobs that cannot be practiced from home, military and health care employees (B −0.104, *P-value* < 0.05), had a personal history of contact with COVID-19 patients (B 0.053, *P-value* < 0.05), higher educational levels (B 0.052, *P-value* < 0.05), and a positive attitude toward taking a vaccine (B 0.088, *P-value* < 0.05). Depression risk was significantly higher in men, non-UAE nationals, in those with lower knowledge scores, and younger ages. The most followed practices were staying home, handwashing, avoiding social gatherings, limiting three people per vehicle, and avoiding public transportation. The least practiced measures were covering the face while sneezing or coughing and wearing masks. Although staying home was reported by 92.5% of participants, 22.6% mentioned that they were visited by more than two people and visited others in 18.4% during the last week. Social media was the source of information for 82.1% of the participants and most trusted doctors and healthcare providers. Depression risk was present in 18.9% of the participants, and most respondents (89%) agreed that SARS-COV-2 infection would be finally be successfully controlled. An encouraging finding is the willingness of two-third of the participants (64.5%) to take the COVID-19 vaccine and if it was developed, although it was very early in the pandemic. Only 14.6% said they would not take the vaccine, and 20.9% were not sure. The obtained results on knowledge and practices, although satisfactory, could be insufficient to prevent this pandemic from being contained. Therefore, we recommend the intensification of awareness programs and good practices. In addition, mental health is an area worth further studies.

## Introduction

The emerging SARS-COV-2 infection pandemic that started at the end of 2019 in Wuhan, China, has tremendously affected the life of the global population. No event in recent history had such widespread impact (1–3). Although most people infected with SARS-COV-2 have mild to moderate disease, people who have underlying comorbidities might develop a more serious life-threatening illness; moreover, due to its high infectivity, the gravely affected patients have crippled healthcare systems across the world (1). The UAE was affected significantly. The total number of cases reached more than a half-million cases by mid-2021 (4). Nevertheless, the UAE response and mitigation efforts were among the best globally (5, 6), with a death rate of 0.29%, among the lowest in the world, and the UAE COVID-19 vaccination coverage reaching 50% of the total population in mid-2021.

Although new treatments and COVID-19 vaccines are being developed and approved via Emergency Use Authorization (EUA) worldwide to mitigate the impact of the pandemic (7). Nevertheless, the population informed choices regarding preventive measures are still essential as the time scale for vaccine coverage world wide and the continuous emergence of new varients of concern influences the timeline of pandemic end (8).

The disease was contained in the city of its origin mainly through population mass isolation and social distancing, with strict strategies to limit the infection rate among the population (9). The world health organization (WHO) and the United States Center for Disease Control and Prevention (CDC) released recommendations to control the spread of the infection, which were rapidly implemented by many governments with variable success rates. The focus was on improving the population's infection control measures. These measures were extended to total lockdown in most countries in the world, as in the UAE, US, Europe, and many other countries. The measures were extreme with a stay-at-home directive, closed schools, and canceled sporting and cultural events (2). The COVID-19 virus high transmissibility, even among presymptomatic patients who may spread the infection without knowing, makes these extreme measures the only option for containment (4, 5, 9). Therefore, studying the knowledge, attitude, and practices of the population regarding virus transmission is a determinant step in the containment efforts of the pandemic. The present study aims to assess these aspects, knowledge, attitude, and practice, toward COVID-19 in the United Arab Emirates among the adult population and identify its determinants to design intervention strategies for an effective prevention program.

## Materials and Methods

### Study Design

This community-based cross-sectional survey assessed the knowledge, attitude, and practices toward infection by SARS-COV-2.

### Target Population and Sampling Procedure

Data was collected during 4–14th April 2020 over a period of 10 days. In total, 1,992 people responded to the survey, 1,867 of which were included in the analysis as the others were from outside the UAE. Assuming a precision of 5% and a 95% confidence level, the minimum required sample size should have been 1,056, and our number was in accordance to this requirement. Convenient sampling was possible owing to the use of an electronic survey distributed through social media (WhatsApp, Instagram, and Snapchat applications). The following inclusion criteria were applied: people over 16 years old and living in the United Arab Emirates.

### Study Instrument

At the time of the study design, there was no validated questionnaire for SARS-COV-2 infection; therefore, a new questionnaire was designed and structured based on previous studies assessing knowledge, attitude, and practices toward previous outbreaks (6, 10). The questionnaire was further adjusted to accommodate the emerging SARS-COV-2 infection using available data obtained in a literature review (11). The questionnaire was constructed to cover five main domains: 1. demographic data; 2. knowledge assessment; 3. practice assessment; 4. attitude assessment; and 5. patient health questionnaire-2 (PHQ-2) as a screening tool for depression among participants (Appendix 1).

#### Demographics

Demographics included; age, gender, nationality, marital status, educational level, occupation, city, and whether they or a family member/friend had been diagnosed with COVID-19.

#### Knowledge Assessment

To measure the knowledge toward SARS-COV-2 infection, we used eight multiple-choice questions with answers including the “I don't know” option and one open-ended question about safe social distancing. Questions developed using the information obtained in the literature review were used to measure knowledge regarding the causative agent, transmission, symptoms, high risk groups, and prevention and precaution measures. The accumulative score was the resultant of adding all correct answers. Higher scores thus indicated better knowledge on these aspects. A score of 13 (75% of all questions) was considered good level of knowledge.

A set of 10 questions adapted from previous studies was used to measure the preventive practices. Participants were asked to respond to these questions on a five-point Likert-like scale indicating the frequency of practicing the presented statements, ranging from “always” to “never” with always indicating a better practice. The assessment of participants' practices evaluated the following behaviors: handwashing, use of hand sanitizer and face masks, keeping a safe social distance, covering mouth and nose during sneezing and coughing, using the non-dominant hand whenever in public, avoiding face touching and going out, avoiding the use of public transportation, limiting up to 3 people per vehicle, and avoiding family gatherings and social events. A score of 30 (75% of the questions correct) or higher reflected better adherence to safe practice, good practice. It means that the practice is between most of the times and always.

We elaborated more on panic shopping by asking 2 questions: (1) have you done extra grocery shopping beyond your personal needs after hearing about the spread of SARS-COV-2? (2) How long do you have enough groceries for? Focusing on social contacts made in the previous week, we asked the following: how many times did you go out during the last week? How many times did you visit someone at their home during the last week? How many people visited you last week? How many delivery or maintenance workers visited your home during the last week? Finally, the attitude toward SARS-COV-2 infection was measured by asking if the participant was willing to take the vaccine when available. Information sources and the participants' trust in their information resources were assessed.

### Risk Assessment

Personal history of recent travel or contact with COVID-19 cases were investigated as these aspects could be the determinants of the study aim. General health and comorbidities were also investigated as they can influence individual knowledge, attitude, and practices. The questionnaire included questions on conditions like pregnancy, cardiovascular disease, hypertension, diabetes mellitus, cancer, and chemotherapy and use of immunosuppressants. According to the risk assessment questions, we classified respondents in low-risk and high-risk groups (Appendix 2). The resultant questionnaire was pilot tested for content and face validity among 30 participants, and minor revisions were made.

The questionnaire was revised by three family physicians from the family medicine residency program and the head and an internist in the occupational/internal medicine department in Ambulatory Healthcare Services. The study investigators reviewed the developed questionnaire for content and clarity. Changes were made as needed. The questionnaire was developed first in English, and then the final version was translated into the Arabic languages by two bilingual investigators. Back translation was done by another two bilingual investigators. Finally, the questionnaire was distributed among a small group, 30 participants from the community, for face validity and clarity.

### Data Collection

The final questionnaire, both questionnaire Arabic and English versions, were distributed to the community by a Survey Monkey (www.surveymonkey.com) link sent through social media. Participation was completely voluntary. This questionnaire distribution method was found suitable for reaching large numbers of people in the community, and also for avoiding close personal contact and preventing COVID-19.

### Ethical Consideration

This research was approved by the Ambulatory Healthcare Services' Human Ethics Committee and the Abu Dhabi Healthcare Services Central Human Ethics Committee. Consent was obtained from all participants as it was a pre-requisite to start the survey. The first page of the survey as well included information about the study.

### Statistical Analysis

Statistical analysis was performed using SPSS statistics version 23 (IBM, Armonk, USA). Demographic data are expressed as numbers and percentages. The knowledge and practice scores were calculated from participants' responses to the KAP statements. The practice questions as wearing a mask and social distancing were presented in percentages.

To identify factors associated with better knowledge attitude or practice a multivariate linear regression analysis, stepwise, was used. It included all explanatory variables available for the whole sample. Associations are presented as regression constant and standard error. Regression coefficients with *P* < 0.05 were deemed statistically significant.

## Results

The study had an excellent response rate with most of the samples collected within 3 days. We obtained a sample of 1,882 people, considering 1,904 who completed the survey and those excluded for being from outside the UAE and under 16 years of age. In the final sample, the mean age of participants was 36.0 years S.D. 10.8. More than two thirds 1,261 (67.7%) were younger than 40 and 602 (32.3%) were older than 40 years old. Males were 360 (19.3%) and most respondents were female 1,510 (80.7%). UAE nationals were the majority of respondents 1,471 (78.2%) and most of them were from Abu Dhabi 1.179 (63.6%). Government employees were 814 (44.8%), 181(9.9)% were healthcare worker, 479 (26.2%) and 259 (14.2%) were students. The majority of participants, 1,604 (87.7%), were working from home, students, or unemployed at the time of the questionnaire. Approximately half the respondents (1,018, 54.5%) held a bachelor's degree or above. Three quarters of respondents were healthy (1,380, 73.3%), with no underlying medical conditions (Table 1).

**Table 1:** Demographic characteristics of participants and knowledge score by demographic variables.

**Characteristics**	**Variables**	**Number (%)**
Nationality	UAE	1,471 (78.2)
	Non-UAE	411 (21.8)
Gender	Male	360 (19.3)
	Female	1,510 (80.7)
Emirates	Abu Dhabi	467 (24.8)
	Dubai	144 (7.7)
	Sharjah	133 (7.1)
	Umm Al-Quwain	137 (7.3)
	Ajman	113 (6)
	Ras Al-Khaimah	93 (4.9)
	Fujairah	65 (3.5)
	Al Ain (AD)	730 (38.8)
Age Group	Under 20	182 (9.8)
	21–30	406 (21.8)
	31–40	673 (36.1)
	41–50	436 (23.4)
	51–60	141 (7.6)
	61–70	25 (1.3)
Education	Less than high school	104 (5.6)
	High school	348 (18.6)
	Diploma	186 (10)
	Bachelor's degree	1,018 (54.5)
	Master or higher	211 (11.3)
Work	Government	814 (44.5)
	Police and defenses	45 (2.5)
	Healthcare	181 (9.9)
	Manual Labor	23 (1.3)
	Business	29 (1.6)
	Student	259 (14.2)
	Unemployed	479 (26.2)
Work duty	Healthcare or defenses or police	226 (12.3)
	Home-based jobs	1,604 (87.7)
Risk Score^*^	No Risky Health condition	1,380 (73.3)
	One condition	404 (21.5)
	Two conditions	76 (4)
	Three conditions	17 (0.9)
	Four conditions	5 (0.3)
Total		1,882 (100)

* *Risk score: number of risky health condition such as; pregnancy, diabetes, hypertension, smoking, cardiovascular disease, asthma or COPD, active cancer or chemotherapy, use of immunosuppressant medication, e.g., steroids*.

### Knowledge

Among all respondents, 1,498 (79.6%) answered 76.5% of the questions correctly (13/17). The mean SARS-COV-2 infection knowledge score was 11.5 (standard deviation, SD 2.5), and only 344 (18.3%) scored <10. The knowledge score was significantly determined by higher educational levels (B 0.18, *P*-value < 0.05), good preventive practice (B 0.121, *P*-value < 0.05), and higher risk scores (B 0.053, *P-value* < 0.05) (Table 2). Interestingly, only half 94 (52.2%) of the healthcare providers correctly answered the recommended social distance of 2 meters; this result does not differ much from the non-healthcare workers' participants. Only 799 (47%) of the non-healthcare workers knew the right distance.

**Table 2:** Determinants of knowledge attitude and practice using regression analysis.

**Dependant variable**	**Studied factors**	**Beta**	**Standard error**	***P*-value**
Knowledge	Education	0.165	0.054	<0.001
	Practice score	0.12	0.014	<0.001
	Risk score	0.053	0.092	0.025
Practice	Non-health or military-police job	−0.104	0.316	<0.001
	Age	0.097	0.01	<0.001
	Knowledge score	0.086	0.043	0.001
	Will take vaccine?	0.088	0.138	<0.001
	Nationality	−0.08	0.248	0.001
	Have contacted COVID-19 patient	0.053	0.479	0.028
	Level of education	0.052	0.1	0.042
Socialization	Male Gender	4.984	0.188	<0.001
	Higher level of education	1.111	0.053	0.05
	Keeps more grocery supply at home	0.987	0.005	0.005
	Recent travel	1.253	0.104	0.03
	PHQ-2	1.067	0.035	0.062
	Age	1.463	0.055	<0.001
	Practice score	0.945	0.013	<0.001
Attitude: time frame for pandemic to end	Agreement to the statement that the Pandemic can be controlled.	−0.253	0.52	<0.001
	Have contacted COVID-19 patient	0.074	0.812	0.002
	Non-health or military-police job	−0.061	0.524	0.011
Attitude: vaccine acceptance	Male gender	0.063	0.045	0.01
	People who wear masks	0.1	0.013	<0.001
	Age	−0.102	0.002	<0.001
	Knowledge score	0.066	0.007	0.008
	UAE Nationality	0.066	0.045	0.008
Trust in social media	Age	0.988	0.006	0.063
	Male Gender	0.73	0.153	0.04
	PHQ-2	1.508	0.186	0.027
	Practice score	0.961	0.017	0.016
	UAE Nationality	1.864	0.143	<0.001
	Higher level Education	0.844	0.066	0.01
Visited by others	Knowledge	−0.08	0.008	0.001
	Age	−0.067	0.002	0.005
Adherence to wearing face masks	Age	0.15	0.003	<0.001
	Non-health or military-police job	−0.13	0.1	<0.001
	UAE Nationality	−0.112	0.081	<0.001
	Vaccine acceptance	0.086	0.045	<0.001
	Male Gender	0.055	0.083	0.019
PHQ-2 score	Higher level of education	0.503	0.148	0.008
	Practice score	0.947	0.006	<0.001
	Risk score	0.943	0.024	0.017
Participants' trust in doctors	UAE Nationality	1.409	0.145	<0.001
	Age	0.973	0.006	0.022
	Higher education level	0.756	0.067	0.026
	PHQ2 depression risk	1.037	0.182	0.031
	Knowledge score	1.019	0.026	0.007
	Non-home based jobs	1.87	0.167	<0.001
	(health, defense and police)			

### Practice

The survey uncovered important determinants of the population with regards to choices and adherence to safe practices in the pandemic. Although excellent adherence was reported, with more than 1,694 (90%) reporting staying home, 536 (28.5%) and 116 (6.2%) reported having visited or been visited by someone. In addition, 1,118 (59.4%) received a delivery worker in their homes at least once. Two thirds, 1,255, of the respondents achieved score of 78.9% (31 points out of 38) on the best practices in preventing SARS-COV-2 infection, which is a good level of practice. The mean score on SARS-COV-2 infection prevention practices was 29.5 (SD 4.2). Respondents who were more likely to adhere to better practices were those of older age (B 0.097, *P*-value < 0.05), with higher knowledge score (B 0.086, *P*-value < 0.05), were of non-UAE nationalities (B −0.08, *P*-value < 0.05), with jobs that cannot be practiced from home, military and health care employees (B −0.104, *P*-value < 0.05), had a personal history of contact with COVID-19 patients (B 0.053, *P*-value < 0.05), higher educational levels (B 0.052, *P*-value < 0.05), and a positive attitude toward taking a vaccine (B 0.088, *P*-value < 0.05) (Table 2).

People who were more likely to socialize and not stay at home during the pandemic were male (B 1.6, *P* < 0.05), with higher education levels (B 0.105, *P* < 0.05), had a smaller grocery supply (B −0.013, *P* < 0.05), had personal history of recent travel (B 0.225, *P* < 0.05), scored higher in the PHQ-2 questionnaire (B 0.065, *P* < 0.05), were older in age (B 0.381, *P* < 0.05), and presented poor scores on prevention practices (B −0.057, *P* < 0.05). Results showed that people who were more likely to visit others were of younger age (B −0.067, *P* < 0.05) and had inferior knowledge on the pandemic (B −0.086, *P* < 0.05) (Table 2).

**Figure 1:**
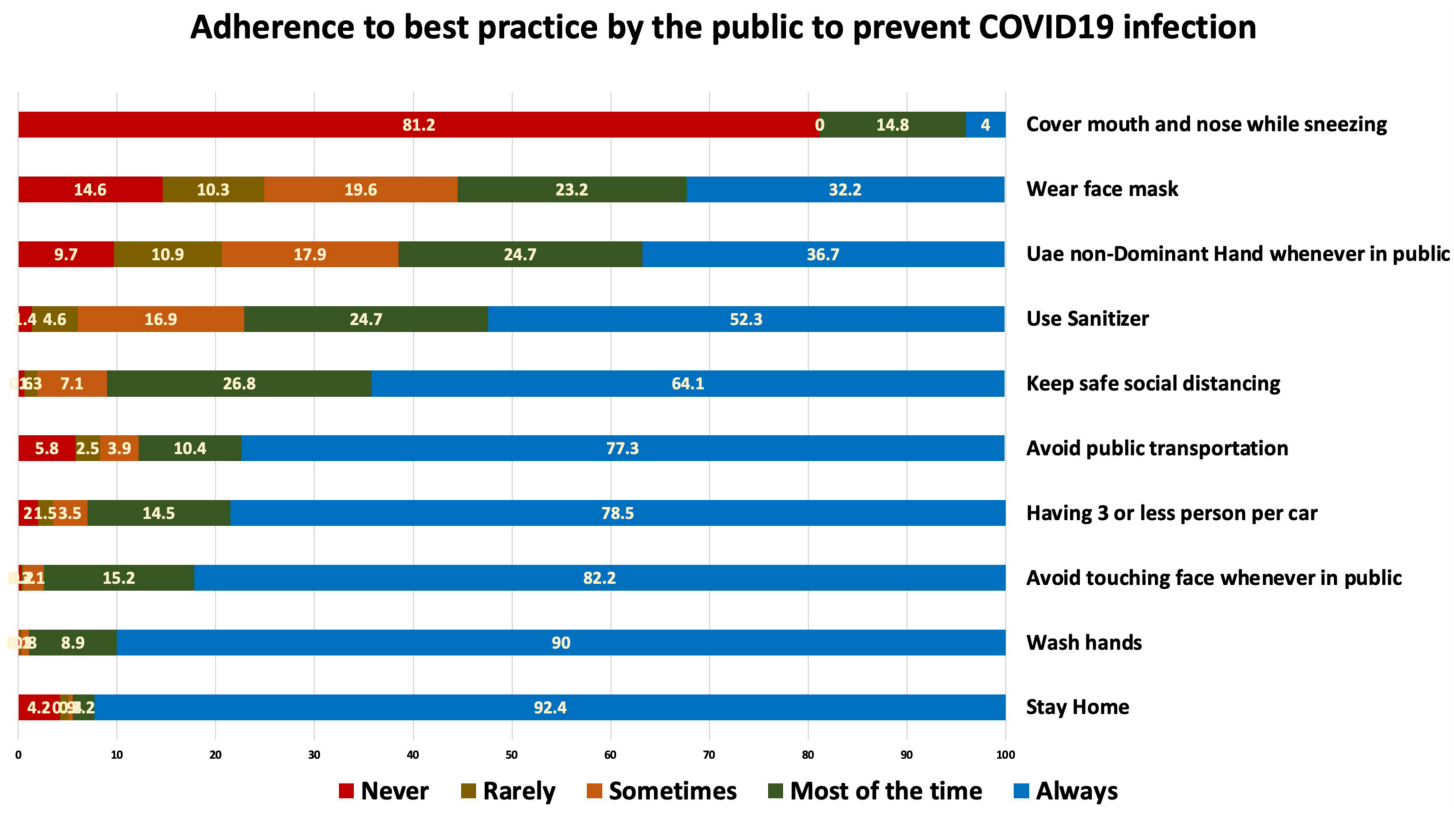
COVID-19 preventive practices reported by the surveyed population.

Wearing face masks in public is considered one of the most important measures to prevent transmission of this infection (12). Nevertheless, only one third 606 (32.2%) of the participants reported always wearing a mask (Figure 1). People who were compliant to wearing masks were more likely to be older in age (B 0.15, *P* < 0.05), work in health or military fields (B 0.13, *P* < 0.05), be non-UAE nationals (B −0.112, *P* < 0.05), have a positive attitude toward taking a vaccine (B −0.086, *P* < 0.05), and be male (B 0.055, *P* < 0.05) (Table 2).

Among the participants, 181 (9.9%) were healthcare workers. It is important to highlight their practices, where 125 (69%) reported always keeping social distancing, 81 (44.8%) always wearing a face mask, 170 (93.9%) always washing hands, and 133 (73.5%) always using hand sanitizer.

### Attitude

The majority of the respondents 1,675 (89%) agreed that COVID-19 will finally be successfully controlled; 706 (37.5%) believed that this pandemic would end within 2–3 months. Participants' beliefs in optimistic time frames to control the pandemic depended on the nature of their work area: more optimism was perceived among healthcare workers and military (B −0.061, *P*-value < 0.05) and less optimism was shown by those with personal history of contact with COVID-19 patients (B −0.074, *P*-value < 0.05) (Table 2).

People who were more likely to accept a vaccine wore masks (B 0.01, *P*-value < 0.05), were of younger age (B −0.102, *P*-value < 0.05), non-UAE nationals (B −0.08, *P*-value < 0.05), had higher knowledge scores (B 0.066, *P*-value < 0.05), and were male (B 0.063, *P*-value < 0.05) (Table 2).

### Trust and Sources of Information

Half the participants trusted doctors and healthcare providers as sources of information (56.7%). Using linear regression, these participants were younger in age (B −0.015, *P* < 0.05), UAE nationals (B 0.627, *P* < 0.05), with lower educational levels (B −0.149, *P* < 0.067), had higher knowledge scores (B 0.07, *P* < 0.05), scored higher in risk of depression (B 0.393, *P* < 0.05), and worked in non-homebased jobs such as healthcare, police, or army forces (B 0.954, *P* < 0.05).

Regarding the respondents' sources of information, social media was the source of information of 1,545 (82.1%) participants. Television news came next as a source of information for 1,001 (53.2%) participants and doctors werre reported as a source by 573 (30.4%). Those using social media as a source of information were more likely to be UAE nationals (B 0.627, *P* < 0.05), younger in age (B −0.015, *P* < 0.05), with a lower educational level (B 0.149, *P* < 0.05) and a higher risk of depression (B 0.393, *P* < 0.05), with high knowledge scores (B 0.07, *P* < 0.05), and were more likely to be military or health care employees (B 0.954, *P* < 0.05).

### Mental Health During the Pandemic

Almost one in five 356 (18.9%) participants presented a depression risk, which was assessed using a PHQ-2 questionnaire (Appendix 3). We observed that depression risks were significantly associated with lower knowledge scores (B −0.059, *P* < 0.05), younger age (B −0.055, *P* < 0.05), and non-UAE nationality (B −0.0395, *P* < 0.05) (Table 2).

## Discussion

The study showed a good knowledge level, positive attitude, and acceptable practices toward SARS-COV-2 infection. There are gaps identified that are of concern, but the UAE population showed comparable levels to other parts of the world. A recent systematic review and meta-analysis on knowledge, attitudes, and practices of the general population about COVID-19. In the review, 89.5% of the studies reported good knowledge, 100% reported a positive attitude, and 93.2% reported satisfactory practice (13).

In this study almost 80% of the respondents answered correctly to 76% of the knowledge questions, indicating that most respondents were knowledgeable about SARS-COV-2 infection. This study was conducted during the early to intermediate stage of the COVID-19 pandemic, April 2020, this level of good knowledge is expected due to increased population's interest in information rergarding SARS-COV-2 and the perceived impact of the pandemic on participants lives. However, there are still areas of concern, such as social distancing (half of the respondents did not know the correct safe distance between people) which may not have had enough focus among the public by awareness campains.

Since knowledge was associated with higher educational levels and 75.8% of participants held a diploma degree or higher, this may explain the good knowledge level of the participants, and more focus needs to be given to groups in the community with lower educational levels. Our study also showed a significant positive association between the level of knowledge and a participant's risk score, where assessment of the risk depended on the presence of medical conditions that lower the participant's immunity, making them more susceptible to being infected (2).

The significant positive association between level of education and SARS-COV-2 infection knowledge score supports this speculation. A Chinese study has found a similar high knowledge rate among its participants (90%), indicating that the Chinese population is knowledgeable about SARS-COV-2 (14). However, according to Haque et al. (10), among 2,045 respondents across Bangladesh, only 54.87% had higher knowledge score regarding COVID-19. This study showed that knowledge on SARS-COV-2 infection among Bangladeshi people depended significantly on age, gender, education level, place of residence, income, and marital status (15). An important finding in this study is that knowledge scores determine both practice and attitude. This finding was similar to a large recent study that reported significant correlations between all KAP scores (16). This finding highlight the importance of knowledge to change behavior and practice and justify investment in effective educational programs. Similar to recent studies, this study participants would actively learn about the infection from the internet and social media as information resources (17).

Regarding practices, the majority of participants reported staying at home (92.8%), which was expected considering the national awareness programs and the adherence to the sanitization campaigns across the Emirates. The same applies to aspects such as washing hands and avoiding touching one's own face. Although high knowledge was associated with better preventive practices, the adherence to some practices such as covering the face while sneezing and coughing (never = 81.2%) and wearing a mask (always or sometimes = 55.4%) was suboptimal. This is much less than a study in neighboring Saudia Arabia. The majority of participants in that study (76.2%) had good habits, including the commitment to hand hygiene, cough etiquette, and limiting their social interaction (18).

The most concerning result is probably the lack of face mask use. When evaluating the current literature, we observed that although there are no available randomized controlled trials (RCTs) that prove the effectiveness of mask wearing on preventing SARS-COV-2 transmission, studies report that a critical practice to reduce transmission of a respiratory viral infection is the use of face masks, surgical masks, or N95 respirators (15). Initial Chinese preventive recommendations and public practice guidelines highlighted the use of face masks for protection against SARS-COV-2 (19). These recommendations are reflected by the results obtained by Zhong et al. (20), where 98% of the respondents in China reported wearing a face mask when going out (14). A study performed in India showed that only 76% of the respondents used face masks (14). Considering the UAE population, we highlight the need for a stronger adherence to the prevention measures, espicialy wearing masks, in order to control the current pandemic.

Until good vaccines' coverage and effectiveness are reached, the only available public health tools to control person-to-person transmission are isolation and quarantine, social distancing, and community containment measures (6). Our study results showed a lack of adherence to home quarantine, since 81.6% of the respondents visited someone and 77.4% were visited in the previous week, which was during the national wide quarantine period. Arab culture and extended family relations in the region can explain the poor adherence to important precautionary measures such as social visits. When comparing our results to those obtained in China, Zhong et al. (20) reported better practices, where 96.4% of the participants had not visited crowded places and 98% wore masks when going out in recent days (20). Difference in adhereance to wearing mask between China (20), and UAE might be attrubitted to the fact that UAE particepants are living in less crowded cities and have large extended families who they visit often.

With regards to attitude, most of the participants of our study (70.67%) held an optimistic attitude toward the COVID-19 pandemic: 37.50% believed that it would finally be successfully controlled within 2–3 months, while 33.17% thought that it would be controlled in <2 months. More optimistic findings were observed among Chinese people, where 90.8% believed that COVID-19 would finally be successfully controlled, and 97.1% were confident that China could control the virus (20). Nevertheless, this study participants were far more optimstic than a recent study participants that included 71 890 individuals from 22 countries where >50% were uncertain or not optimistic that the pandemic will finally end or that their government will be able to control COVID-19 situation (16). These findings can be explained by the trust in government authorities, the local healthcare system, and the country's infrastructure by UAE residents. In this study, people who believed that infection control would take longer tended to be non-healthcare workers, military, police officers, and those who had contact with people suffering from SARS-COV-2 infection.

This study found a deprerssion risk among the participants of 18.9%, a value that represents twice what was reported last year among ambulatory health services patients (10%) (12). A study assessing the relationship between depression risk and the SARS-COV-2 pandemic found that in a population-based study, being female and a student, as well as having symptoms suggestive of COVID-19 and poor perceived health were associated with higher rates of anxiety and depression. On the other hand, the availability of accurate information and the use of specific preventive measures, such as handwashing, seemed to mitigate these effects (14). In this study, better knowledge was also protective against depression risk, highlighting an aspect of depression prevention that could be focused by strong awareness programs, especially among the young who showed a higher depression risk in this study.

Unexpectedly, the practices of UAE healthcare workers as assessed by our study were suboptimal. A study performed in Nigeria reported low knowledge (78.6%) and poor attitude (64%) among healthcare workers (14). On the contrary, Saqlain et al.'s (21) findings showed that Pakistani healthcare workers had good knowledge (93.2%, *n* = 386), positive attitude (8.43 ± 1.78), and good practices (88.7%, *n* = 367) regarding SARS-COV-2. Similar findings by Giao et al. (22) illustrated that the majority of participants among healthcare workers in Vietnam held good knowledge and good attitude toward SARS-COV-2. More research is needed to investigate healthcare workers' competences and the knowledge necessary for their work in this challenging pandemic environment.

### Strengths and Limitations

The strength of this study relies in its large sample size, recruited during a short period of time in the middle stage of the SARS-COV-2 pandemic in the UAE. The UAE is one of the countries with the highest access to internet and use of social media, thus an online survey, offered in Arabic and English languages, was able to reach wide sections of the society. The interval of distribution was 10 days only, other UAE studies with similar design recruited double this study sample size when conducted over 2 months (23). But many were of similar sample size (24) and compared to studies done elsewhere as in the US the reach of the online survays in the UAE is excellent (25). Nevertheless, when comparing our results to the most recent national population statistics of the UAE, our sample was over-representative of women, well-educated people, and young adults. Age of the particepnts might be affected by the mean by which the questionnaire was distributed since most of social media users are young adults. Overrepresentation by female sex and young populations is found as well in other online-based studies and worth investigating with regards to casuses and its influence on results (23, 24).

We spaculate that the presence of positive attitude toward COVID-19 may also differ depending on socioeconomical status. Vulnerable populations such as the elderly and manual labor workers could have been underestimated in our sample, the age group 60 years and over, were 25, and manual workers were 23. The older age group is most at risk of complications if getting the infection. While the manual labor workers are a higher risk group to get the infection in any community and undermine the country containment efforts due to the nature of their work. Their work necessitates being in close contact with different groups consistently and their educational and Knowledge level may be less than the average community members. Therefore, more suitable data collection methods are needed as interviews. Another limitation is that the survey was only translated to Arabic and English, underestimating the participation of speakers of other languages. Additionally, a limitation of our study is the development of an objective assessment tool of practices toward SARS-COV-2 infection. Due to the very limited time for developing our questionnaire, we measured practices by simple self-reported questions only. As well there was no well-developed validated questionnaire to be used from previous studies due to the nature of the COVID-19 pandemic being caused be new virus. Nevertheless, future studies can be done as a follow up study and to further validate the tool.

Future research is well-needed to study effective interventions that improve population knowledge attitude and practice with rergards to the implementation of evidence based effective protective measures such as face masks, staying home and vaccination.

## Conclusion

The UAE residents with higher education levels, good practices, and higher risk scores have good knowledge about SARS-COV-2 infection and satisfactory practices and attitude, which suggests that health education programs should aim to improve the population's adherence to the safe practices. With the size and impact of this pandemic, lack of adherence even by a minority could be enough to prevent the pandemic from being contained. The recommendations obtained from this study are the intensification of awareness programs and interventions, and an increase in good practices. Moreover, the COVID-19 pandemic has negatively affected the community's mental health, therefore more attention should be dispensed on the psychiatric impact of this condition and healthcare providers should consider tackling this issue when evaluating their patients.

## Data Availability Statement

The raw data supporting the conclusions of this article will be made available by the authors, without undue reservation.

## Ethics Statement

The studies involving human participants were reviewed and approved by Abu Dhabi Department of Health Human Ethics Committee. The patients/participants provided their written informed consent to participate in this study.

## Author Contributions

LB, HA, AA, and RA participated in conceptualization and writing of the manuscript. LB analyzes data and revised manuscript. All authors listed have made a substantial, direct and intellectual contribution to the work, and approved it for publication.

## Conflict of Interest

The authors declare that the research was conducted in the absence of any commercial or financial relationships that could be construed as a potential conflict of interest.
