# Supplementary material for: Knowledge, Attitude, and Practices Toward SARS-COV-2 Infection in the United Arab Emirates Population: An Online Community-Based Cross-Sectional Survey
Source: Front Public Health. 2021 Jul 19;9:687628. doi: 10.3389/fpubh.2021.687628 (PMC8326444; doi:10.3389/fpubh.2021.687628)
Supplement: Supplementary file 1 [file Presentation_1.pdf]

Appendix 1. Knowledge, attitude, and practices towards SARS-COV-2 among UAE residents: questionnaire:

**Questionnaire of knowledge towards SARS-COV-2 INFECTION:**	
Questions	Answer options
K1. Corona (SARS-COV-2 INFECTION) is considered:	Bacteria, gas, virus, fungus, mosquito bite, unknown, I don't know
K2. A person may have Corona (SARS-COV-2 INFECTION) if he/she has the following (choose all that apply):	Fever, cough, sneezing, no symptoms, I don't know
K3. Corona (SARS-COV-2 INFECTION) virus is spread by (Choose all that apply):	Air, droplets, touching hard surfaces, food, I don't know
K4. The following are effective measures to decrease the possibility of getting Corona (SARS-COV-2 INFECTION)? (Choose all that apply)	Drinking extra water, hand washing, using hand sanitizer, wearing a mask, taking a multivitamin supplement, I don't know
K5. How many of those infected with Corona (SARS-COV-2 INFECTION) will recover?	One third of them, half of them, more than 90% of them, I don't know
K6. It is not necessary for children and young adults to adopt measures to prevent Corona (SARS-COV-2 INFECTION) infection as they are not at high risk of death or complications?	True, false, I don't know
K7. If isolation is required, it should be for how long?	One week, two weeks, three weeks, four weeks, I don't know

K8. People returning from travel should avoid going out in public and adhere to home quarantine for at least how long?	One week, two weeks, three weeks, four weeks, I don't know
K9. How many meters are considered a safe social distance between you and others?	(No option, one answer)
**Questionnaire of practices towards SARS-COV-2 INFECTION:**	
Questions	Answer options
P1. During last week, how often did you wash your hands?	Always, most of the time, sometimes, rarely, never
P2. During last week, how often did you use hand sanitizer?	Always, most of the time, sometimes, rarely, never
P3. During last week, how often did you use a face mask?	Always, most of the time, sometimes, rarely, never
P4. During last week, how often did you keep social distancing?	Always, most of the time, sometimes, rarely, never
P5. During last week, how often did you cover your mouth and nose if sneezing or coughing?	Always, most of the time, sometimes, rarely, never
P6. During the last week, how often did you use your non-dominant hand whenever in public?	Always, most of the time, sometimes, rarely, never
P7. Did you avoid touching your face (mouth, nose, and eye) whenever in public?	Always, most of the time, sometimes, rarely, never
P8. During the last week how often did you stay home and avoid going out unless for necessary purposes?	Always, most of the time, sometimes, rarely, never

P9. During the last week how often did you avoid using public transportation and being in crowded places?	Always, most of the time, sometimes, rarely, never
P10. During the last week how often did you have 3 people or less per vehicle during transportation?	Always, most of the time, sometimes, rarely, never
P11. During the last week how often did you avoid family gatherings and other social events?	Always, most of the time, sometimes, rarely, never
P12.Have you done extra grocery shopping beyond your needs after hearing about the spread of the Corona (SARS-COV-2 INFECTION) infection?	□Yes □No □Sometimes
P13. For how long do you have enough groceries for?	□One Day □3 Days □One Week □2 Weeks □3 Weeks □One Month □More than one month
P14. How many times did you go out last week?	
P15. How many times did you visit someone's home last week?	
P16. How many people visited you last week?	
P17. Do you stay at home during the time of the national sanitization program (8:00 pm to 6:00 am)?	□Yes □No □Sometimes
P18. How many meters are considered a safe social distance between you and others?	
**Questionnaire of attitude towards SARS-COV-2 INFECTION**	
Questions	Answer options

1. What is your resource of Corona (SARS-COV-2 INFECTION) information? (Choose all that apply)	Newspaper; TV; radio news; social media including Twitter, Instagram, or Whatsapp; official websites including WHO and CDC webpages; other people; other resources.
2.Which of the following is the most reliable source in your opinion? (Choose all that apply)	Newspaper; TV; radio news; social media including Twitter, Instagram, or Whatsapp; official websites including WHO and CDC webpages; other people; other resources.
3.Do you agree that Corona (SARS-COV-2 INFECTION) infection will finally be successfully controlled?	Yes, no, somehow, I don’t know<br>If yes when? 1 week, 2 weeks ,1 month, 2-3 months, 4-6 months, >6 months
4.If the vaccine arrives, I will be willing to get vaccinated.	□Yes □No □Not sure

Appendix 2. Risk assessment score.

**Any of the following conditions:**	
Pregnancy<br>Diabetes<br>Hypertension<br>Smoking<br>Cardiovascular disease<br>Asthma or COPD<br>Cancer or chemotherapy<br>Use of immunosuppressant medication, e.g. steroids<br>Other (specify)	Score 1 point for each condition.<br>The total risk score is the sum of scored points.
None	0

COPD: chronic pulmonary obstructive disease.

Appendix 3. Patient Health Questionnaire-2 (PHQ-2).

Over the last 2 weeks, were you bothered by any of the following:				
	Not at all	On several days	On more than half the days	Nearly every day
Little interest or no pleasure.				
Feeling down, depressed, or hopeless				
